# Encapsulating peritoneal sclerosis—a rare but devastating peritoneal disease

**DOI:** 10.3389/fphys.2014.00470

**Published:** 2015-01-05

**Authors:** Zia Moinuddin, Angela Summers, David Van Dellen, Titus Augustine, Sarah E. Herrick

**Affiliations:** ^1^Department of Transplantation, Manchester Royal InfirmaryManchester, UK; ^2^Faculty of Medical and Human Sciences, Institute of Inflammation and Repair, University of Manchester, Manchester Academic Health Science CentreManchester, UK

**Keywords:** encapsulating peritoneal sclerosis, peritoneal dialysis, mesothelium, epithelial–mesenchymal transition, fibrosis

## Abstract

Encapsulating peritoneal sclerosis (EPS) is a devastating but, fortunately, rare complication of long-term peritoneal dialysis. The disease is associated with extensive thickening and fibrosis of the peritoneum resulting in the formation of a fibrous cocoon encapsulating the bowel leading to intestinal obstruction. The incidence of EPS ranges between 0.7 and 3.3% and increases with duration of peritoneal dialysis therapy. Dialysis fluid is hyperosmotic, hyperglycemic, and acidic causing chronic injury and inflammation in the peritoneum with loss of mesothelium and extensive tissue fibrosis. The pathogenesis of EPS, however, still remains uncertain, although a widely accepted hypothesis is the “two-hit theory,” where, the first hit is chronic peritoneal membrane injury from long standing peritoneal dialysis followed by a second hit such as an episode of peritonitis, genetic predisposition and/or acute cessation of peritoneal dialysis, leading to EPS. Recently, EPS has been reported in patients shortly after transplantation suggesting that this procedure may also act as a possible second insult. The process of epithelial–mesenchymal transition of mesothelial cells is proposed to play a central role in the development of peritoneal sclerosis, a common characteristic of patients on dialysis, however, its importance in EPS is less clear. There is no established treatment for EPS although evidence from small case studies suggests that corticosteroids and tamoxifen may be beneficial. Nutritional support is essential and surgical intervention (peritonectomy and enterolysis) is recommended in later stages to relieve bowel obstruction.

## Introduction

Encapsulating peritoneal sclerosis (EPS) is a chronic clinical syndrome of insidious onset, presenting late as chronic malnourishment with signs and symptoms of intermittent, acute or sub-acute gastrointestinal obstruction (Augustine et al., 2009). It appears to be a multifactorial disease with several initiating factors that are significant at the different stages of the disease. Diagnosis is confirmed by macroscopic and/or radiological observation of sclerosis, calcification, peritoneal thickening or encapsulation of the intestines (Kawaguchi et al., 2000). EPS can be fatal but, fortunately, it is a rare complication predominantly of long-term peritoneal dialysis (PD). Since the first case of EPS was reported in 1980 (Gandhi et al., 1980), there has been a steady rise in the incidence of the disease from 0.9% in 1996 to 3.3% in 2005 as patients stay on PD for longer and awareness of this complication has increased. The data obtained over the years from several retrospective studies performed to investigate this potentially fatal condition estimate the worldwide incidence of EPS at 0.7–3.3% in patients on PD (Brown et al., 2009). The mortality rate for patients with EPS is great at 25–55%, predominately in the year after diagnosis and is directly proportional to the duration of PD treatment (Rigby and Hawley, 1998; Kawanishi et al., 2004; Kawanishi and Moriishi, 2005; Brown et al., 2009).

## Aetiology

EPS is usually seen in end-stage renal disease (ESRD) patients who have been on long-term PD therapy. Dialysis fluid is damaging to the peritoneum due to the high glucose concentration and acidic pH. A high glucose concentration facilitates the osmosis and diffusion gradient across the peritoneum and the low pH acts to prevent the formation of glucose degradation products (GDPs) which are damaging agents (Jorres et al., 1992). Heat sterilization of PD fluid leads to the formation of GDPs (Wieslander, 1996) and these in the presence of glucose cause the formation of advanced glycation end products (AGEs) (Mortier et al., 2002). More biocompatible solutions are now being used that contain less GDPs which results in reduced peritoneal damage (Boulanger, 2008).

Interestingly, EPS can also develop in patients not on PD but associated with other conditions such as autoimmune diseases, sarcoidosis, peritoneal and intra-abdominal malignancies, chronic peritoneal ascites, intra-peritoneal chemotherapy, intraperitoneal exposure to particulate matter or disinfectant, abdominal surgery, endometriosis, intra-peritoneal infections (tuberculosis), and beta-blocker administration (Pollock, 2001; Kawanishi and Moriishi, 2005) (Table 1). Chronic renal failure itself could also induce peritoneal changes including thickening before PD therapy (Williams et al., 2002) and uremia, a contributory factor in all PD patients, is known to lead to a pro-inflammatory state (Baroni et al., 2012).

**Table 1:** **Non-peritoneal dialysis related causes of EPS**.

• Autoimmune diseases
• Sarcoidosis
• Peritoneal and intra-abdominal malignancies
• Chronic peritoneal ascites
• Intra-peritoneal chemotherapy
• Intraperitoneal exposure to particulate matter or disinfectant
• Abdominal surgery
• Endometriosis
• Intra-peritoneal infections (tuberculosis)
• Beta-blocker administration

## Risk factors

The duration of PD therapy seems to be the most important risk factor for the development of EPS. In an Australian survey, the incidence of EPS increased with the duration of PD, being 1.9, 6.4, 10.8, and 19.4% in patients on PD for more than 2, 5, 6, and 8 years respectively (Rigby and Hawley, 1998).

A Japanese prospective study reported similar findings, in which the incidence of EPS was 0.7% after 5 years, 2.1% after 8 years, 5.9% after 10 years, and 17.2% after 15 years of PD therapy (Kawanishi et al., 2004). A more recent Scottish study also reported an increase in incidence of EPS from 2% at 2–3 years to 8.8% at 5–6 years of PD therapy (Brown et al., 2009). However, most cases of EPS are diagnosed after discontinuation of PD therapy. A Japanese prospective study reported that 69% of EPS cases occurred after discontinuation of PD therapy (Kawanishi et al., 2004). This suggests that despite PD being a major risk factor for EPS, lavage of the peritoneal cavity during PD may possibly limit the accumulation of factors that encourage the development of the disease (Yamamoto et al., 2005, 2010).

Interestingly, organ transplantation appears to increase the risk of developing EPS as some studies have reported a high incidence shortly after renal transplantation (Fieren et al., 2007; Balasubramaniam et al., 2009). This could either be due to the acute cessation of PD or due to the profibrotic effect of immunosuppressive medication (calcineurin inhibitors). Calcineurin inhibitors, like Tacrolimus and Cyclosporin, cause up-regulation of transforming growth factor-beta (TGF-β), and other profibrogenic factors potentiating matrix accumulation (Khanna et al., 2002). In experimental rat models, Cyclosporin combined with chronic peritoneal exposure to dialysis solution was associated with increased peritoneal fibrosis and angiogenesis (Van Westrhenen et al., 2007). However, the exact mechanism of post-transplantation EPS still remains unknown.

Peritonitis is a common complication of PD and plays a complex part in the development of EPS with the number of peritonitis episodes linked to incidence of EPS (Yamamoto et al., 2005). Recurrent peritonitis due to bacterial contamination including *Pseudomonas* spp., *Staphylococcus aureus*, and certain fungal organisms have been particularly implicated in the development of EPS (Flanigan et al., 1984; Chew et al., 1997; Rigby and Hawley, 1998). Other risk factors suggested to be involved in EPS onset are the composition of dialysis fluid and generation of GDPs, young age, ultrafiltration failure and the exposure to PD catheter cleaning reagent, chlorhexidine (Pollock, 2001) (Table 2).

**Table 2:** **Risk factors for EPS in the peritoneal dialysis (PD) population**.

• Duration of PD
• Acute cessation of PD
• Organ transplantation
• Peritonitis
• Composition of dialysis fluid (low pH, high glucose)
• Young age
• Ultrafiltration failure
• Exposure to chlorhexidine

## Clinical features of EPS

Patients usually present with abdominal symptoms like early satiety, anorexia, nausea, vomiting, and altered bowel habit (constipation or diarrhea in the early stages of EPS) (Nakamoto, 2005; Augustine et al., 2009). These symptoms may be accompanied by signs of inflammation (pyrexia and raised CRP) and/or blood stained ascites in the early stages (Nakamoto, 2005; Maruyama and Nakayama, 2008). Late stages of EPS are associated with abdominal pain, fullness, overt bowel obstruction and presence of an abdominal mass). This is caused by the development of a fibrous cocoon that gradually covers the intestines and leads to malnutrition, weight loss, bowel obstruction, ischemia and strangulation, infection and death (Kawaguchi et al., 2000).

## Classification of EPS

Based on the clinical presentation, Nakamoto categorized EPS into four groups (Nakamoto, 2005). The following are the proposed clinical stages:

*Stage 1- Pre-EPS stage*: Asymptomatic with mild ascites and no inflammation.
*Stage 2- Inflammatory stage:* Patients are symptomatic with nausea and diarrhea consistent with partial encapsulation of the bowel and intestinal swelling. Mild inflammation with fibrin exudation is present.
*Stage 3- Encapsulation:* Symptoms of bowel obstruction due to the formation of the fibrous cocoon causing encapsulation. It can be associated with mild to severe inflammation.
*Stage 4- Chronic stage of ileus:* Patients have absolute bowel obstruction caused by thickening of the encapsulating fibrous cocoon. There is little, if any, inflammation at this stage.

## Diagnosis of EPS

The diagnosis of EPS relies on clinical findings, radiological tests and pathological appearance of the diseased tissue. Clinically, the diagnosis of EPS is based on recognizing the signs and symptoms (nausea, anorexia, early satiety, weight loss, altered bowel habit, and ascites) in the patients at risk of developing the condition (Nakamoto, 2005). Blood tests may also reveal high CRP and low albumin levels. These presenting symptoms and signs are vague and non-localizing. However, the insidious nature and chronicity of development can be a distinguishing feature of EPS (Kawaguchi et al., 2000). As a consequence, EPS is often not recognized in its early stages and requires a high index of suspicion to pursue a diagnosis.

**Figure 1:**
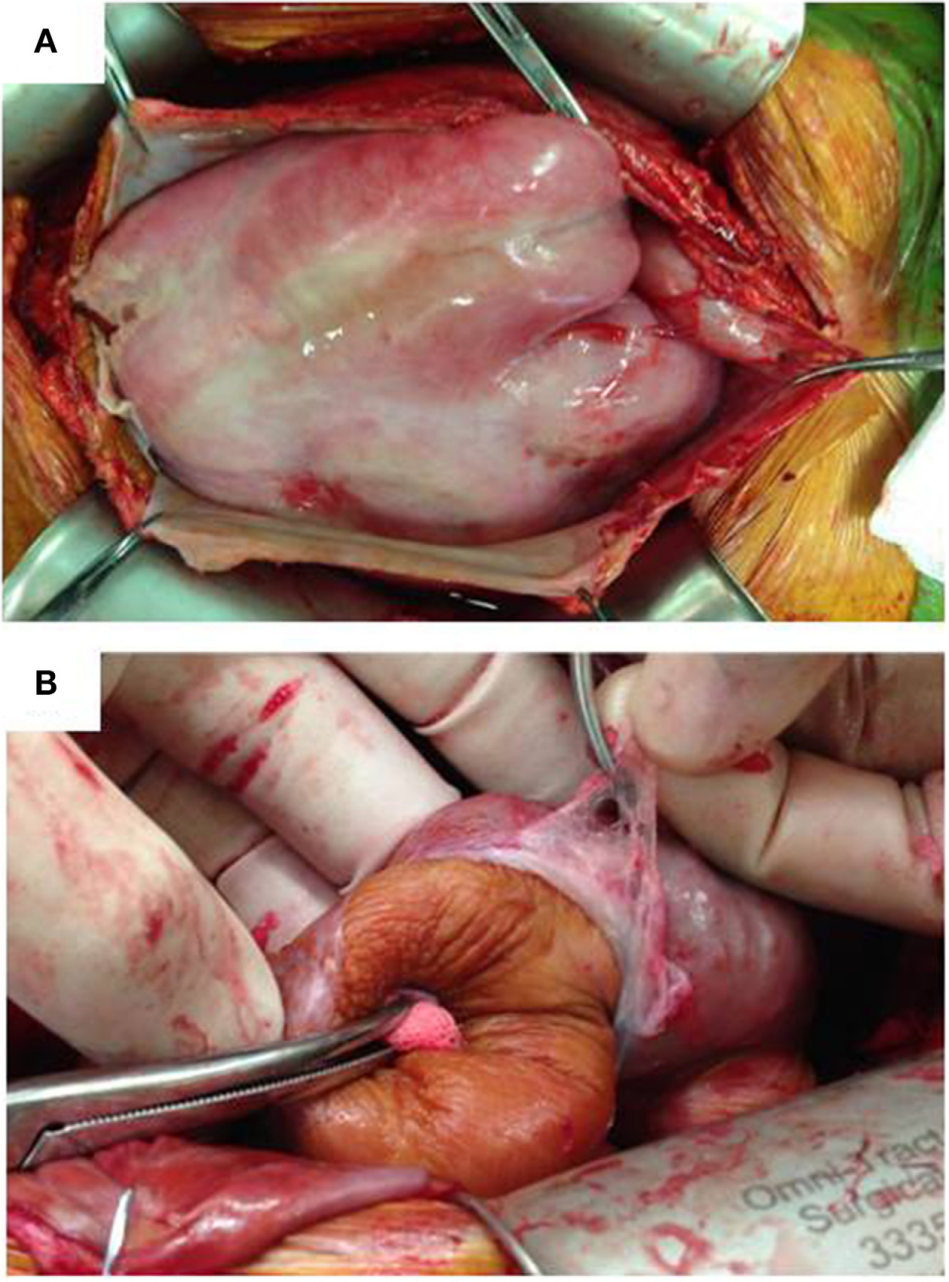
**Macroscopic appearance of EPS at surgery**. **(A)** Thickened parietal peritoneum is held up by surgical clips. Visceral peritoneum is thickened forming a fibrous cocoon encapsulating the bowel. **(B)** Visceral peritoneum peeled off the small bowel which has a brownish, tanned leathery appearance.

As the clinical picture of EPS can vary considerably, various investigations are necessary to further evaluate a suspected case (Kawaguchi et al., 2005). Ultrasonography, water-soluble contrast studies and computed tomography (CT) scanning are the most widely used radiological tests to aid the diagnosis of EPS. CT scanning, however, is the investigation of choice in patients with established EPS and helps monitor disease progression. Peritoneal enhancement, peritoneal thickening, calcification, bowel tethering, bowel wall thickening, signs of bowel obstruction, and loculated collections are the most common CT findings of EPS (Vlijm et al., 2009). But, given the rare, complex nature of the disease and with most of the CT scan appearances being non-specific, interpretation and diagnosis can be difficult. Surgery (Laparoscopy/Laparotomy and peritoneal biopsy) therefore may be needed to confirm the diagnosis (Kawaguchi et al., 2005). At surgery, macroscopically, advanced cases of EPS typically exhibit a thickened brownish peritoneum with a cocoon-like encapsulation of the entire intestine by the visceral peritoneum (Figure 1). The intestinal loops are adherent to one another and the visceral peritoneum is severely thickened with fibrosis. Adhesions between the visceral and parietal peritoneum are rare, except in cases of severe inflammation (Honda and Oda, 2005). Macroscopic evidence of peritoneal calcification is also a feature but is not necessarily present in all cases of EPS (Park et al., 2008).

Numerous studies have been performed searching for potential biomarkers in PD effluent to aid the early diagnosis of EPS. Low CA125 levels (denoting mesothelial cell loss) and high levels of the inflammatory cytokine Interleukin-6 (IL-6) in PD effluent have been noted several years before the diagnosis of EPS suggesting that these factors could be potential early diagnostic markers (Sampimon et al., 2010). However, further research is necessary to identify definite biomarkers for early diagnosis of EPS. Current imaging techniques are also not useful at reliably detecting EPS in its early stages. CT scan screening of asymptomatic PD patients is not recommended as EPS may occur within a year of a normal CT scan. Also, not all long-term PD patients with peritoneal abnormalities on CT go on to develop established EPS (Goodlad et al., 2011). Dynamic cinematographic magnetic resonance (cine-MR) scanning with advanced image analysis may be useful in early detection of EPS. However, more studies are required to validate the efficacy of this imaging modality (Wright et al., 2011). Laparoscopy performed in suspected cases may have an important role to play in the early diagnosis of EPS. Our current lack of understanding of the pathophysiology limits our ability to detect EPS early or prevent its occurrence.

## Histological features of encapsulating peritoneal sclerosis

The two most relevant pathologies of long-term PD are simple sclerosis and EPS. Simple sclerosis is characterized by the thickening of parietal peritoneum in the absence of encapsulation. The uremic state and continuing PD leads to a gradual loss of mesothelium (mesothelial denudation). Medial sclerosis and hyalinization of the peritoneal vasculature (vasculopathy) is also seen along with neoangiogenesis (Honda et al., 1996). The sub-mesothelial compact zone thickens and is composed of myofibroblasts and fibrous collagen (Mateijsen et al., 1999). In addition, AGEs are found to be present in the mesothelial and sub-mesothelial layer of PD patients (Yamada et al., 1994; Nakayama et al., 1997). Of note the amount of angiogenesis was found to be proportional to the severity of intestinal fibrosis (Mateijsen et al., 1999; Plum et al., 2001) and the level of mesothelial deudation correlated with sub-mesothelial thickening and vasculopathy (Williams et al., 2003). Simple sclerosis is a fairly common finding in long-term PD patients while EPS is rare. Therefore, studies analysing the histological differences in the peritoneum from EPS and simple sclerosis patients are limited. Of these, only one study performed by Garosi et al. (2005) analyzed both visceral and parietal peritoneum, and Braun and colleagues analyzed only visceral peritoneum (Braun et al., 2012) while the remaining studies did not specify the type of peritoneum analyzed (Table 3). Garosi and colleagues investigated 180 peritoneal biopsies of PD patients with simple sclerosis and compared morphological findings with those from biopsies of 39 patients with EPS (Garosi et al., 2005). Significant findings in patients with EPS were thickening of the sub-mesothelial cell layer, vasculopathy, arterial occlusion, inflammation, tissue and arterial calcification, and ossification. In a similar study, fibrin deposition, increase in the size of fibroblasts, capillary angiogenesis, and mononuclear cell infiltration were more common features of EPS than simple sclerosis (Honda et al., 2003). Sherif and colleagues compared the peritoneum of EPS with simple sclerosis and showed that the sub-mesothelial compact zone was thinner in early-EPS than late-EPS (Sherif et al., 2008). In a more recent study, fibroblast-like cells, mesothelial denudation, calcification, decreased cellularity and positive iron staining were more common in the peritoneum of EPS patients. Positive immunohistochemical staining for podoplanin, a lymphatic endothelial marker expressed by peritoneal mesothelial cells, was significantly more prevalent in EPS peritoneum (Braun et al., 2012). Up-regulation of vascular endothelial growth factor (VEGF) and down-regulation of mast cells (Alscher et al., 2007; Braun et al., 2009) also appeared to be a feature of EPS peritoneum. However, as the above findings are not always specific to EPS tissue, reliable histological diagnosis is difficult. Further studies are therefore necessary to explore the presence of additional unique immunohistochemical markers to aid the diagnosis.

**Table 3:** **Comparative histopathology of EPS patients and non-EPS PD patients**.

	Braun et al., 2012	Braun et al., 2009	Sherif et al., 2008	Garosi et al., 2005	Honda et al., 2003
EPS patients (*n*)	31	9	12	39	12
Non-EPS PD patients (*n*)	27	10	30	180	57
Visceral (V) or parietal (P)	–	V	–	V and P	–
**Histologic parameters**	**Significance between EPS and non-EPS PD patients**				
Fibrosis	S	S	S	–	NS
Degenerated layer thickness	–	–	S	–	–
Fibroblast like cells	S	–	–	–	–
Inflammation	NS	–	–	S	S
Mesothelial denudation	S	–	NS	–	NS
Vasculopathy	NS	S	–	S	NS
Fibrin deposition	S	–	S	–	S
Vessel density	NS	S	–	–	–
Fe deposits	S	–	–	–	–
Decreased cellularity	S	–	–	–	–
Fibroblast enlargement	–	–	–	–	S
Capillary angiogenesis	–	–	–	–	S
Calcification	NS	–	–	S	–

*(S) and (NS) denote significance and non-significance, respectively. (–) denotes data not being measured or recorded*.

## Pathophysiology of encapsulating peritoneal sclerosis

Although a number of risk factors have been identified, there are currently no means of preventing EPS development or recognizing earlier stages of the disease. Furthermore, it is a matter of debate whether EPS is a natural progression of simple sclerosis or a completely separate entity. However, it is clear that having simple sclerosis does not predispose to developing EPS. It is generally now accepted that a two-hit theory explains EPS development. It is well established that chronic exposure to bio-incompatible dialysate causes damage to the peritoneum of all patients on PD. This first hit causes mesothelial disruption which can trigger a fibrotic process which is referred to as “simple sclerosis.”

**Figure 2:**
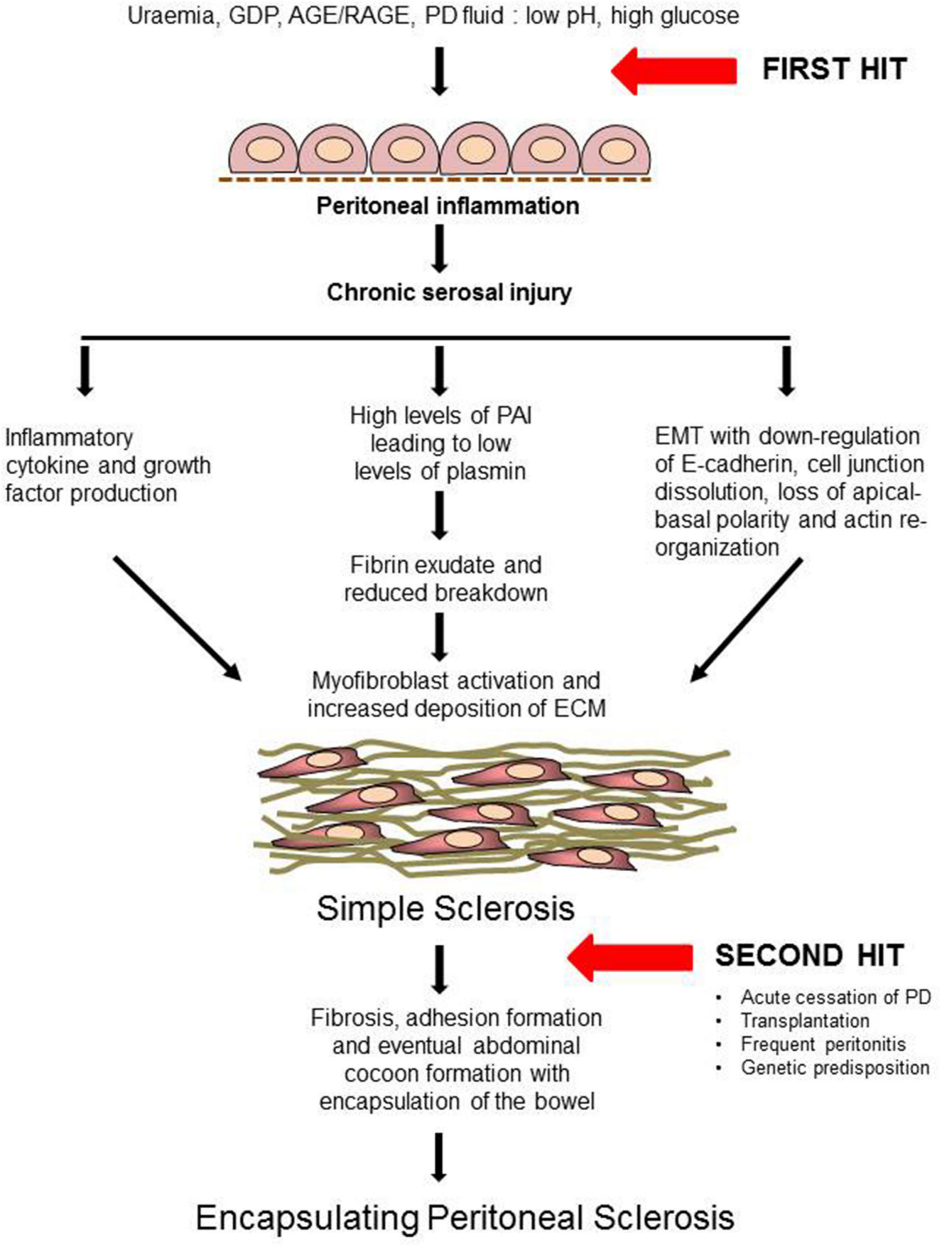
**Proposed pathogenesis of EPS: “Two-hit” theory (Modified from Augustine et al., 2009)**. AGE, Advanced Glycation End-products; RAGE, Receptors for Advanced Glycation End-products; IL-1, Interleukin-1; TNF, Tumor Necrosis Factor; TGF-β, Transforming Growth Factor-β; VEGF, Vascular Endothelial Growth Factor; EMT, Epithelial to Mesenchymal Transdifferentiation; MMP, Matrix Metalloproteinases; ECM, Extracellular Matrix.

The subsequent exposure to a second insult, in some patients, triggers the development of EPS. This can be either an episode of peritonitis, acute cessation of PD, transplantation, any other acute intra-abdominal event or maybe a genetic predisposition (Figure 2). A number of genetic polymorphisms with functional effects have been described in patients on PD and these may partially explain the propensity to develop EPS (Pletinck et al., 2012). A number of rodent models of EPS have been developed using chronic chemical irritation to induce peritoneal sclerosis and bowel encapsulation (Hoff, 2005; Summers et al., 2008). Such models replicate the cycles of tissue injury with fibrin deposition and inflammation leading to subsequent repair with mesothelial cell and fibroblast activation, proliferation and matrix deposition that are proposed to drive the development of EPS. Some of these mechanisms of induction are described in more detail below.

### Inflammatory profile

The PD catheter can itself trigger inflammation either directly as a foreign material or by acting as a site of bacterial biofilm formation leading to PD peritonitis (Flessner et al., 2007). Various characteristics of PD fluid such as low pH, high glucose levels, hyperosmolarity, presence of GDPs, and AGEs promote the release of several growth factors such as Transforming Growth Factor-β (TGF-β) and Platelet Derived Growth Factor (PDGF) and pro-inflammatory cytokines, IL-1, IL-6, IL-18, and Tumor Necrosis Factor-alpha (TNF-α) potentiating fibrosis (Margetts and Bonniaud, 2003; Baroni et al., 2012).

Clinical studies have found elevated levels of IL-1β, IL-6, IL-8, TGF-β1, Hepatocyte Growth Factor (HGF), and PDGF in ascites from EPS patients in comparison with non-EPS controls (Masunaga et al., 2003). As EPS develops, patients also suffer from subclinical bowel ischemia which may cause translocation of bacteria across the bowel wall into the peritoneal cavity leading to infection, further inflammation and fibrosis (Augustine et al., 2009).

### Fibrin deposition and fibrinolysis

Peritoneal inflammation initiates fibrin exudation that can either be lysed or remodeled by invading fibroblasts leading to fibrosis and adhesion formation (Holmdahl, 1997; Sulaiman et al., 2002). Plasmin plays a key role not only in fibrin degradation but also in the turnover of extracellular matrix and activation of matrix metalloproteinases. Plasmin is formed from the inactive zymogen plasminogen under the influence of plasminogen activators: tissue plasminogen activator (tPA) and urokinase-like plasminogen activator (uPA), whereas, plasminogen activation inhibitors (PAI-1 and PAI-2) reduce plasminogen activation. Low levels of plasmin and high levels of PAI were found in serum of patients on PD compared with hemodialysis (HD) patients suggesting a higher degree of hypercoagulation (Tomura et al., 1996). Mesothelial cells express PAI-1 and 2 under the influence of TGF-β (Rougier et al., 1998; Holmdahl et al., 2001) and such an inappropriate balance between fibrin deposition and breakdown increases the probability of fibrous adhesion formation possibly contributing to the development of EPS.

### Epithelial–mesenchymal transition (EMT) of mesothelial cells

In terms of peritoneal fibrosis, substantial evidence points to mesothelial cells (MCs) as being the principle source of myofibroblasts via a process of epithelial–mesenchymal transition (EMT) (Aroeira et al., 2007). MCs are unique in their expression of both epithelial and mesenchymal markers, and when exposed to injurious agents, they lose cell-cell contact and apical-basal cell polarity and invade the basal lamina changing to a mesenchymal phenotype expressing α-smooth muscle actin and depositing extracellular matrix (Aguilera et al., 2005). EMT occurs during normal embryo development and tumor cell invasion and metastasis and is a complex process that requires the correct spatiotemporal expression, interaction and modification of a multitude of intra-and extracellular factors to allow a change in cell phenotype (Thiery et al., 2009). Loss of cell surface E-cadherin is a prerequisite for EMT and primarily controlled by three main families of transcription factors: zinc finger Snail, basic helix-loop-helix, and ZEB (Kalluri and Weinberg, 2009). Several groups have shown the presence of mesothelial markers (ICAM-1 and cytokeratin) localized with spindle-shaped fibroblast- like cells in the sub-mesothelial layer in peritoneal biopsies from patients undergoing PD suggesting EMT had occurred (Yanez-Mo et al., 2003; Jimenez-Heffernan et al., 2004). Mesothelial cells isolated from dialysis fluid effluents from patients undergoing peritoneal dialysis also undergo a transition from epithelial to mesenchymal with a loss of E-cadherin (Zhang et al., 2013) and induction of snail (Yanez-Mo et al., 2003). Furthermore, AGE products (De Vriese et al., 2006) and dialysate (Selgas et al., 2006) induce human mesothelial EMT in culture. The process of EMT can occur under the influence of a number of pro-inflammatory and profibrotic cytokines, however TGF-β is proposed to be a principle mediator of mesothelial EMT as demonstrated both *in vitro* (Hung et al., 2003; Yang et al., 2003) and *in vivo* (Margetts et al., 2005). In terms of therapeutic strategy, blocking TGF-β1 (Loureiro et al., 2011), or addition of tamoxifen (Loureiro et al., 2013a) bone morphogenic protein-7 (Yu et al., 2009), or microRNA30a (Zhou et al., 2013) have all been found to protect the peritoneum from dialysate—induced damage in experimental models. Although strong evidence suggests that mesothelial EMT is important for the development of peritoneal sclerosis, the role this process plays in EPS, is a matter of debate (Loureiro et al., 2013b).

### Growth factors potentially involved in the development of EPS

The pathways involved in the development of EPS are likely to be complex and involve the interaction of a number of important growth factors leading to sub-mesothelial thickening and cocooning of the bowels. The level of the pro-neoangiogenic growth factor, Vascular Endothelial Growth Factor (VEGF), detected in peritoneal effluent has been found to directly correlate with length of time the patients are on PD (Zweers et al., 2001). Furthermore, treatment with VEGF blocking antibody reduced the thickness of the sub-mesothelial layer and reduced vasculopathy in a rat model of EPS (Io et al., 2004) suggesting a key role for this factor in EPS development. VEGF is produced by cultured human peritoneal mesothelial cells in response to various stimuli such as GDPs known to be present in PD fluid (Mandl-Weber et al., 2002). TGF-β, another important growth factor in wound healing and fibrosis, has also been shown to be present in peritoneal dialysate from patients (Lin et al., 1998; Yanez-Mo et al., 2003) Exposure of human mesothelial cells to TGF-β1 induced procollagen type 1 expression (Hung et al., 2003) and in rodent models, TGF-β1 administration caused peritoneal fibrosis (Margetts et al., 2005), and adhesion formation (Williams et al., 1992; Gorvy et al., 2005). Moreover, studies in a hyperglycemia-induced rodent model showed that AGEs upregulate TGF-β and induce submesothelial fibrosis with interstitial accumulation of collagen (De Vriese et al., 2003). Furthermore, intraperitoneal administration of a first generation adenovirus overexpressing TGF-β1 in mice resulted in submesothelial thickening and angiogenesis up to 10 days after administration (Margetts et al., 2005). However, a helper dependent adenovirus subsequently used with longer term expression of TGF-β1 led to encapsulation of the bowels in a thick cocoon in a similar manner to the final stages of EPS (Liu et al., 2009). Over-expression of TGF-β also increased levels of matrix metalloproteinase-2 (MMP-2) and high amounts of this protease found in PD effluent has been considered a potential marker of peritoneal injury and progression to EPS (Hirahara et al., 2007). In a mouse model, inhibition of MMP-2, TGF-β, and VEGF significantly improved peritoneal fibrosis and angiogenesis (Ro et al., 2007). Many other growth factors including HGF, PDGF, Connective Tissue Growth Factor (CTGF), and Fibroblast Growth Factor (FGF), shown to be involved in the development of fibrosis in other organs, may also influence the onset of EPS (Korte et al., 2011a).

## Therapeutic management of EPS

### Discontinuation of PD

Cessation of PD should be done as soon as the diagnosis of EPS has been established. However, as cessation of PD is a known risk factor for the development and progression of EPS, this approach has been under much debate. Regular peritoneal lavage after discontinuation of PD has been tried in Japan with varying results. A recent retrospective study performed by Yamamoto et al. (2010) has demonstrated that regular peritoneal lavage helps mesothelial cell layer recovery and prevention of EPS. However, given the risk of peritonitis with peritoneal lavage and the increased association of peritonitis and prolonged PD therapy with EPS progression, it is best to stop PD at the time of diagnosis of EPS along with removal of the PD catheter (Habib et al., 2011; Kawanishi, 2012).

### Nutritional support

As most patients with EPS suffer from malnutrition, appropriate nutritional support is imperative in the management of these patients. Studies from our centre have demonstrated poor pre-operative nutritional status and improved survival with peri-operative Total Parenteral Nutrition (TPN) in patients with advanced EPS who underwent surgery (peritonectomy and enterolysis) (De Freitas et al., 2007; Campbell et al., 2014). However, TPN therapy on its own does not have any curative effect as demonstrated by a study from Japan which showed no recovery in patients treated with TPN alone (Kawanishi and Moriishi, 2005). TPN therapy is therefore best used as an adjunct to more definite treatment in EPS patients with malnutrition or peri-operatively until gut function recovers.

### Medical treatment

The evaluation of the efficacy of pharmacological treatments for EPS is difficult due to the lack of good evidence based data. However, the treatment options currently in vogue are as follows:

#### Immunosuppressive therapy

Corticosteroids are the drugs of choice in the management of EPS. They are particularly useful in the treatment of EPS in its early inflammatory stages (Kuriyama and Tomonari, 2001; Kawanishi, 2012). Steroids possibly act by suppressing inflammation, preventing fibrin deposition and collagen synthesis and maturation (Habib et al., 2011). Steroids also help in preventing the formation and reducing the accumulation of ascites (Mori et al., 1997; Jung and Cho, 2013). Various case reports and series have reported good prognosis and improved survival with the use of steroids compared to any other immunosuppressive agent (Junor and McMillan, 1993; Rajani, 2002). However, in a study from Japan, the rate of clinical improvement with isolated steroid treatment was only 38.5% (Kawanishi et al., 2004). Nevertheless, steroids are effective in treating the early stages of EPS but the clinical response tends to reduce in the later stages that are associated with increased fibrosis and bowel obstruction. A variety of other immunosuppressive medications like azathioprine, rapamycin, mycophenalate mofetil, sirolimus, and cyclosporine have also been used to treat EPS either alone or in combination with steroids (Rajani, 2002; Wong et al., 2005; Lafrance et al., 2008). However, apart from steroids, evidence regarding the efficacy of any other immunosuppressive therapy in EPS remains weak due to lack of robust randomized clinical trials (Bozkurt et al., 2009).

#### Tamoxifen

Tamoxifen is a Selective Estrogen Receptor Modulator (SERM) with antifibrotic properties and has been used in the treatment of various fibrotic disorders like retroperitoenal fibrosis, fibrosing mediastinitis, fibrosing cerivicitis, and desmoid tumors (Cornelis and Oreopoulos, 2011). Various case reports and small series have reported satisfactory outcomes following the use of Tamoxifen in the treatment of EPS (Guest, 2009; Cornelis and Oreopoulos, 2011). A recent large retrospective Dutch study has demonstrated significantly reduced mortality in EPS patients that were treated with Tamoxifen (45.8%) when compared to those that were not treated with Tamoxifen (74.4%) (Korte et al., 2011b). Tamoxifen appears to exert its effect through inhibition and modulation of TGF-β. *In vitro* and animal models showed that Tamoxifen treatment blocked EMT induced by TGF-β, preserved the fibrinolytic activity and reduced the migration capacity of mesothelial cells leading to reduced fibrosis and reduced PD effluent levels of VEGF leading to reduced angiogenesis in the peritoneum (Loureiro et al., 2013a). However, Tamoxifen has almost always been used in combination with corticosteroids, therefore the efficacy and safety of Tamoxifen alone in the treatment of EPS still remains to be evaluated. The potential side-effects of Tamoxifen (deep vein thrombosis, endometrial cancer, and calciphylaxis) also need to be considered (Del Peso et al., 2003). Further prospective trials are therefore necessary to establish the safety and efficacy of Tamoxifen in the treatment of EPS.

### Surgery

Surgical techniques in the management of EPS have evolved over the last decade. Surgery is routinely performed in patients with advanced EPS in Japan and in the UK at specialist referral centers by surgeons experienced in the management of EPS (Augustine et al., 2009; Kawanishi, 2012). Surgery is usually performed in the late stages of EPS, in patients that present with absolute bowel obstruction or as surgical emergencies with an acute abdomen. Currently favored surgical techniques are peritonectomy and careful enterolysis which involves resection of the peritoneum and fibrous tissue together with division of adhesions to release the bowel (Figure 1). Mortality post-surgery ranges from 19 to 34.5% (Summers et al., 2005; Kawanishi, 2012; Latus et al., 2013) and is mainly seen in patients with advanced EPS presenting as surgical emergencies (Augustine et al., 2009). Although surgery is the most successful form of treatment for patients with advanced disease, recurrence rates post-surgery tend to be high at around 25%. However, in Japan, the recent introduction of Noble plication (suturing of the intestines to each other to prevent obstruction) along with routine enterolysis has reduced the recurrence rate to 12.3% (Kawanishi, 2012). Tamoxifen and steroids may also be continued post-operatively as they may have a role in preventing recurrence of EPS (Lo and Kawanishi, 2009).

## Prevention

Although various studies have demonstrated possible mechanisms in the development of EPS, we still have no strategy to prevent the occurrence of this condition. As the risk of developing EPS increases with the duration of PD, there has been much debate on an “expiry date” for PD in patients. Studies from Japan have suggested 8 years to be a safe period to continue PD beyond which patients should be switched to hemodialysis (Kawanishi et al., 2004). However, setting an expiry date is not recommended as it could have a negative impact on the quality of life and could increase the risk of complications of tunneled lines used for hemodialysis in patients who were symptom free on PD (Garosi et al., 2005). A more rational approach would be to assess peritoneal membrane function. The commonly used method to assess peritoneal deterioration is the Peritoneal Equilibration Test (PET) with a high transport of solutes across the peritoneal membrane being indicative of failing function. There has been a suggestion of cessation of PD in all patients that show a high transport status (Kawanishi, 2012). However, not all patients that are high transporters develop EPS. Therefore, this approach has been under much debate. Peritoneal lavage could potentially be used in patients who have been on long-term PD (>8 years), with high transport status and increased levels of markers of inflammation (IL-6) and low levels of Ca-125 in their PD effluent in order to delay the development of EPS (Moriishi et al., 2002). However, this approach is not universally practiced due to the associated risk of infective peritonitis. Other proposed methods of possible prevention are by using more bio-compatible PD fluid and taking care to reduce the incidence of infective peritonitis. Addition of therapeutic agents such as, Tamoxifen and Angiotensin Converting Enzyme (ACE) inhibitors, may show promise to ameliorate peritoneal membrane function and fibrosis (Kolesnyk et al., 2007). Further studies are therefore necessary to investigate possible therapeutic strategies to prevent the exacerbated fibrotic process which culminates in EPS.

## Conclusion

Despite the increase in incidence and awareness over the last decade, EPS still remains a rare but much feared complication of long-term PD. Prolonged PD therapy is the most important risk factor in the development of EPS. Uremia, inflammation, EMT and loss of mesothelial fibrinolytic response are possible mechanisms of peritoneal fibrosis which could influence the development of EPS. However, the aetiopathogenesis still remains poorly understood. Currently a high index of suspicion in at risk groups is required to make the diagnosis of EPS. CT scan is the investigation of choice to aid the diagnosis, but the use of newer imaging modalities like cine-MRI, that shows promising signs, still needs to be validated. Low levels of Ca-125 and high levels of IL-6 in PD effluent have been suggested as early markers of development of EPS. However, further research needs to be done to validate this claim and look for other potential bio-markers to detect EPS early. The treatment of EPS in its early stages is largely medical with corticosteroids, tamoxifen and nutritional support. However, surgery (peritonectomy and enterolysis) is the treatment of choice for advanced cases presenting with overt bowel obstruction. Peri-operative nutritional support with TPN and post-operative steroids with or without Tamoxifen is recommended to prevent recurrence. Improvement in surgical technique has vastly improved survival of patients with EPS. However, despite successful surgery these patients remain at increased risk of mortality due to ongoing renal replacement therapy. Many clinicians are reticent toward transplantation in these patients due to a perception that transplantation might not be feasible given the complex surgical history. Although, the risk of developing recurrent disease post transplantation exists, the chances of survival are much improved with a functioning kidney transplant. These patients should therefore be assessed and worked up for renal transplantation in order to improve long-term survival.

### Conflict of interest statement

The authors declare that the research was conducted in the absence of any commercial or financial relationships that could be construed as a potential conflict of interest.
